# Role of *Penicillium chrysogenum* XJ-1 in the Detoxification and Bioremediation of Cadmium

**DOI:** 10.3389/fmicb.2015.01422

**Published:** 2015-12-21

**Authors:** Xingjian Xu, Lu Xia, Wei Zhu, Zheyi Zhang, Qiaoyun Huang, Wenli Chen

**Affiliations:** ^1^State Key Laboratory of Agricultural Microbiology, Huazhong Agricultural UniversityWuhan, China; ^2^Northeast Institute of Geography and Agroecology, Chinese Academy of SciencesChangchun, China; ^3^Key Laboratory of Arable Land Conservation (Middle and Lower Reaches of Yangtze River), Ministry of Agriculture, College of Resources and Environment, Huazhong Agricultural UniversityWuhan, China

**Keywords:** *Penicillium chrysogenum*, cadmium, antioxidant system, detoxification mechanism, bioremediation

## Abstract

Microbial bioremediation is a promising technology to treat heavy metal-contaminated soils. However, the efficiency of filamentous fungi as bioremediation agents remains unknown, and the detoxification mechanism of heavy metals by filamentous fungi remains unclear. Therefore, in this study, we investigated the cell morphology and antioxidant systems of *Penicillium chrysogenum* XJ-1 in response to different cadmium (Cd) concentrations (0–10 mM) by using physico-chemical and biochemical methods. Cd in XJ-1 was mainly bound to the cell wall. The malondialdehyde level in XJ-1 cells was increased by 14.82–94.67 times with the increase in Cd concentration. The activities of superoxide dismutase, glutathione reductase (GR), and glucose-6-phosphate dehydrogenase (G6PDH) peaked at 1 mM Cd, whereas that of catalase peaked at 5 mM Cd. Cd exposure increased the glutathione/oxidized glutathione ratio and the activities of GR and G6PDH in XJ-1. These results suggested that the Cd detoxification mechanism of XJ-1 included biosorption, cellular sequestration, and antioxidant defense. The application of XJ-1 in Cd-polluted soils (5–50 mg kg^-1^) successfully reduced bioavailable Cd and increased the plant yield, indicating that this fungus was a promising candidate for *in situ* bioremediation of Cd-polluted soil.

## Introduction

The toxicity of heavy metals to living organisms has attracted considerable attention because some toxic metals can transport and contaminate water and agricultural systems. According to the reports by World Health Organization, cadmium (Cd) is a metal of immediate concern among all hazardous heavy metals (Zafar et al., 2007). The non-degradability of Cd, along with its accumulation and bio-magnification in food chains, increases the toxicity of Cd to biological systems. Therefore, it is necessary to develop remediation strategies for Cd-contaminated soil to protect public health and the environment (Deng et al., 2014). *In situ* immobilization of heavy metals by microbial remediation is more eco-friendly and economically efficient than conventional methods (Siripornadulsil and Siripornadulsil, 2013). Thus, this microbial remediation approach is a good alternative to inhibit the bioavailability of heavy metals to living organisms.

Cadmium exerts a selection pressure on living organisms, including fungi that have developed tolerance mechanisms to withstand high concentrations of Cd. Some fungi can mobilize Cd ions (Gadd, 2001) and consequently affect the biogeochemical mobility and behaviors of Cd in soils. Thus, these fungal features can be exploited to remedy Cd-contaminated soils (Gadd, 2001). For instance, *Glomus mosseae, Glomus versiforme*, and *Glomus diaphanum* reduce Cd uptake through rice shoots (Zhang et al., 2005). Arbuscular mycorrhizal fungi can also effectively ameliorate metal toxicity to plants (Janoušková and Pavlíková, 2010). However, the feasibility of the application of other fungal species, such as *Penicillium chrysogenum*, in heavy metal stabilization was seldom studied. In addition, the fungal remediation mechanisms involved are complex and vary with fungal species and metals (Hartley et al., 1997).

The Cd-resistant mechanisms of fungi were still unclear (Lanfranco, 2007). Cd as a non-redox metal influences cellular redox status by decreasing the pool of the antioxidant glutathione (GSH; Pócsi et al., 2004). Moreover, Cd also causes the following cellular modifications: growth inhibition, alteration of cell morphology, generation of excessive reactive oxygen species (ROS), lipid peroxidation resulting in malondialdehyde (MDA) formation, remarkable interference with DNA repair, and affects the activities of functional enzymes via replacing other metal ions in functional enzymes (Bai et al., 2003; Jaeckel et al., 2005; Xu et al., 2014). The cellular redox status of aerobic organisms is regulated by enzymatic and non-enzymatic defense systems, which eventually decrease the deleterious effects of Cd (Pócsi et al., 2004). GSH is the most abundant intracellular thiol which interacts with heavy metals and participates in ROS sequestration. GSH can be transformed into oxidized glutathione (GSSG). This molecule maintains a redox balance in cells and protects cells from oxidative injury induced by abiotic stresses (Ruiz and Blumwald, 2002). In addition, several enzymes such as superoxide dismutase (SOD) and catalase (CAT) destroy superoxide anion radical ($O_{2}^{–}$) and hydrogen peroxide (H_2_O_2_), respectively (Ott et al., 2002). Meanwhile, glutathione reductase (GR) and glucose-6-phosphate dehydrogenase (G6PDH) are responsible for GSH recycling (Singh et al., 2012). Cd treatment (5 and 50 μM) stimulates SOD activity, GR activity, and GSH level, but exerts no influence on CAT in *Paxillus involutus* (Ott et al., 2002). Cd also strongly induces the enzymes of the sulfur amino acid biosynthetic pathway and enhances GSH synthesis in yeasts (Herrero et al., 2008). These results indicate that GSH is a key factor in eliminating the toxic effects induced by Cd.

In microbial bioremediation, identifying the metabolic and molecular effects of heavy metals and the consequent cellular responses of distinct microbial strains is crucial to understand the different mechanisms involved in the resistance to heavy metals. In our previous studies, we isolated a highly Cd-resistant fungal strain *P. chrysogenum* XJ-1 with a comparable biosorption capacity for Cd (Xu et al., 2012b). The study aims to investigate the effects of Cd on the cell morphology, Cd distribution in different cell compartments, and the antioxidant defense system of XJ-1. Moreover, a pot experiment was also performed to evaluate the remediation effect of XJ-1 in soils contaminated with various levels of Cd (0, 5, 10, and 50 mg kg^-1^ soil) with pakchoi (*Brassica chinensis* L.) in relation to soil Cd fractionations. In the study, we proposed the Cd detoxification mechanism of *P. chrysogenum* XJ-1 at the cellular level for the first time and provided a potential candidate bioremediation agent for Cd-polluted soil.

## Materials and Methods

### Microorganism and Preparation of Fungal Biomass

Wild-type *P. chrysogenum* strain XJ-1 (GenBank accession No. GU733711) was cultured in potato dextrose agar (PDA; Xu et al., 2012b). Streptomycin (final concentration: 100 μg mL^-1^) was added into PDA plates to inhibit bacterial growth. All plates were incubated at 28°C in an incubator for 120 h. Well-grown fungal colonies were preserved in PDA slants. Slant cultures were routinely subcultured every 30 days before experimental use. This strain was maintained in the State Key Laboratory of Agricultural Microbiology, Huazhong Agricultural University, China.

We prepared 7-day-old spores of XJ-1 suspension at a density of 10^6^ spores mL^-1^. Then, 1 mL of spore suspension was inoculated into 250-mL Erlenmeyer flasks containing 100 mL of minimal medium (MM; 5 g of (NH_4_)_2_SO_4_, 15 g of KH_2_PO_4_, 0.6 g of MgSO_4_, 0.6 g of CaCl_2_, 0.005 g of FeSO_4_⋅7H_2_O, 0.002 g of CoCl_2_, and 20 g of glucose per liter of medium, pH 5.0) in the presence of Cd^2+^ at the desired concentration (0, 1, 5, or 10 mM). These metal concentrations were selected on the basis of the sensitivity of XJ-1 to Cd determined in our previous study (Xu et al., 2012b). The inhibition rates of biomass production for XJ-1 were approximately 20, 50, and 60% in various MM with 1, 5, and 10 mM Cd, respectively. Each treatment had three replicates. All culture flasks were incubated at 150 rpm and 28°C for 120 h. Mycelia were harvested by filtration through a Whatman No. 11 filter paper, washed thrice with sterile distilled deionized water to remove residual growth medium, and then divided into multi-subsamples for further analyses. Cultures of the fungus in both media were at the same growth phase and fungal biomass increased at a constant rate. Our preliminary experiment suggested that the mycelia cultures remained in the exponential growth phase for 120 h.

### Cd Uptake by Fungal Strain XJ-1

The quantity of Cd^2+^ absorbed by XJ-1 cells was measured after 120-h exposure to Cd^2+^ at 1, 5, or 10 mM with graphite furnace atomic absorption spectroscopy (F-240, VARIAN) according to the method by Purchase et al. (1997) with minor modifications. The distribution of Cd was measured in different fractions (loosely and tightly bound to cells and absorbed by fungal cells). In brief, fungal cells (120 h) were centrifuged (10000 × *g*, 4 °C, 10 min) to remove residual Cd^2+^, resuspended, and vortexed in 10 mL of distilled water for 10 min. After further centrifugation, Cd^2+^ in this supernatant (loosely bound to cell wall) was determined. Fungal biomass was then suspended in 10 mL of 0.1 M HNO_3_, vortexed for 10 min and centrifuged. Cd^2+^ in such supernatant was equivalent to that tightly bound to fungal cell wall. Finally, the fungal biomass was then suspended in 2 mL of sterile distilled water, digested overnight at 115°C with 6 mL of an equivolume mixture of concentrated H_2_SO_4_ and concentrated HNO_3_. The cooled digested solution was diluted to 10 mL with 1 M HNO_3_ to determine the concentration of Cd^2+^ (equivalent to that accumulated by fungus and not removed by corresponding washing).

### Electron Microscopy Detection

Scanning electron microscopy–energy dispersive X-ray (SEM–EDX) was used to examine the surface of fungal mycelia and determine Cd^2+^ adsorption to cell walls after exposure to 1 mM Cd^2+^ under the conditions mentioned above. The mycelia were freeze-dried overnight to a constant weight, placed on a stainless steel stab with a double-sided tape, and then coated with a thin Pt layer under a vacuum. The samples were observed and the images were digitally recorded under a scanning electron microscope (JSM-6390LV). The voltage was maintained at 30 KeV and the microprobe was focused at 2000 × magnification. Meanwhile, the Pt-coated samples were subjected to EDX spectroscopy. The precipitation of Cd in cells was observed via transmission electron microscopy (TEM). Mycelial samples were fixed through immersion in freshly prepared 2.0% glutaraldehyde in phosphate buffer for 2 h, washed thrice with phosphate buffer (pH 7.0), post-fixed in 1% phosphate-buffered osmium tetroxide for 1 h, and then washed thrice with phosphate buffer (pH 7.0). The samples were embedded in Spurr resin after dehydration with ethanol. Sections (ca. 50–60 nm thick) were measured under an H7650/Hitachi H-700 FA transmission electron microscope.

### Preparation of Cell-free Extracts and Biochemical Determinations

Mycelial samples were collected according to the method as mentioned in Section “Microorganism and Preparation of Fungal Biomass” after 120-h exposure to Cd^2+^ (1, 5, or 10 mM), transferred into a precooled mortar containing liquid nitrogen, and then ground with 0.5 g of sterile glass beads (diameter 1mm) per 1 g of wet weight for mycelia. The resultant paste was suspended in 5 mL of exaction buffer (20 mM Tris, 1 mM EDTA pH 7.5). The homogenate was centrifuged at 8000 × *g* for 10 min at 4°C, and the supernatant was further centrifuged at 15000 × *g* for 20 min at 4°C. The final supernatant was assigned as the cell-free extracts and stored in separate aliquots at -80°C before further analyses. The protein concentration of the cell-free extracts was quantified with the enhanced BCA Protein Assay Kit (P0010S, Beyotime Institute of Biotechnology, Shanghai, China) by using a Synergy HT Multi-Mode Microplate Reader (BioTeK).

Superoxide dismutase, CAT, and GR activities were determined using the Total SOD Assay Kit with WST-1 (S0107), CAT Assay Kit (S0051), and GR Assay Kit (S0055), respectively. G6PDH activity was measured on the basis of the increase in the absorbance at 340 nm due to NADP reduction (Postma et al., 1989). The reaction mixture consisted of 875 μL of deionized H_2_O, 50 μL of 1 M Tris-HCl (pH 8.0), 10 μL of 0.04 mM NADP^+^ (disodium), 10 μL of 0.05 mM MgCl_2_⋅6H_2_O, 50 μL of 0.1 M glucose-6-phosphate, and 5 μL of cell-free extracts. Enzymatic activities were determined after 120-h exposure to Cd and expressed in U mg^-1^ total protein.

Total GSH and GSSG were measured with corresponding assay kits (S0053, Beyotime Institute of Biotechnology, Shanghai, China) and GSH was then estimated from the difference between the total GSH and GSSG. MDA concentration was measured with its corresponding assay kit (S0131). All the assay kits were purchased from Beyotime Institute of Biotechnology, Shanghai, China.

### Pot Experiment

Brown-red soil was collected from the surface (0–15 cm depth) near the Shizi Mountain at Huazhong Agriculture University, China. The sample was air-dried and sieved to 2 mm before analyses. The main physico-chemical parameters of the soil were detected according to the previous method (Chen et al., 2010) as follows: pH, 6.23; organic matter, 8.18 mg kg^-1^; and total Cd, 0.11 mg kg^-1^. Pakchoi (*Brassica chinensis* L.), which was widely grown in China, was selected for this investigation. This plant grows quickly and has an obvious response to bioavailable Cd. Therefore, it can be assigned as an indicator to evaluate the ecological risk of metal-contaminated soils (Chen et al., 2010).

The experiment was conducted in 32 plastic pots containing 1.5 kg of soil in a greenhouse. Soil samples were treated with four levels of CdCl_2_ (0, 5, 10, and 50 mg Cd kg^-1^ soil hereafter denoted as Cd 0, Cd 5, Cd 10, and Cd 50, respectively) and then stored for at least 7 d to equilibrate water in the soil. It should be noted that Cd levels (Cd 0, Cd 5, Cd 10, and Cd 50) were the total Cd concentration in soil, but not the real bioavailable Cd level. The Cd-treated soils in the microbial remediation group were inoculated with the spore suspension of XJ-1. The 32 plastic pots were equally divided into two groups: the control group and the fungal treatment group. Each group included four Cd levels and each Cd level had four replicates. The fungal agent formed by XJ-1 was expressed as F. The spore density of XJ-1 in soil was adjusted to approximately 10^6^ spores g^-1^ dry weight soil. An equal volume of sterile distilled water was sprayed on Cd-treated soils in the control group (CK) without microbial remediation. Ten seeds of pakchoi were sown in each pot and irrigated with water to maintain the water content at approximately 60% during plant germination. The plants were thinned to four per pot, and then the water content was maintained at approximately 20%. Pakchoi was harvested in 3 months after sowing. The plants were collected by cutting at the soil surface and plant yields were examined. The soil was removed from each pot and the roots were removed from the soil. The shoots and roots were washed in 0.01 M EDTA at least three times and then in deionized water to remove non-specifically bound Cd. The plant materials were dried to a constant weight at 70°C with a forced-draught oven. The dried plant materials were weighed and then ground with a stainless steel grinder. Approximately 100 g of soil sample was collected from each pot for subsequent analyses of soil Cd fractions. The water content in each soil sample was determined. The obtained quantitative data were corrected for dry weight estimations.

### Cd Analysis

The plant materials were digested with concentrated nitric acid and Cd was determined with graphite-furnace atomic adsorption spectrometry. A simple sequential extraction procedure (Sposito et al., 1982) was used to fractionate soil Cd into different operationally defined forms, including soluble/exchangeable, organic-bound, inorganic-bound, and residual fractions. Reagent blanks and analytical duplicates were used whenever appropriate to ensure accuracy and precision in the analysis.

### Statistical Analysis

Each experiment was repeated in triplicate or quadruplicate. All data analyses were performed with SPSS 13.0. The data were evaluated by ANOVA and Tukey’s test with the significance level of *p* < 0.05. All plots were conducted with SigmaPlot (version 10.0).

## Results and Discussion

### Cd Distribution and Micromorphology of *P. chrysogenum* XJ-1

As shown in **Table 1**, among various Cd forms, tightly bound Cd in the cell wall of XJ-1 is the highest, followed by intracellular-bound Cd and then loosely bound Cd. The maximum Cd removal by XJ-1 (63.02 mg g^-1^ dry biomass) was obtained in the treatment of 5 mM Cd: 86.21% of tightly bound Cd, 8.46% of intracellular-bound Cd, and 5.33% of loosely bound Cd. In the treatments of 1 and 10 mM Cd, the removal of the tightly bound Cd fraction accounted for 80.77 and 85.24%, respectively. Compared with the treatment of 1 mM Cd, the treatments of 5 and 10 mM Cd significantly increased the proportion of tightly bound Cd, but markedly decreased the fractionation of intracellular-bound Cd. Metal resistance is defined as the ability of an organism to survive metal toxicity through a mechanism generated in direct response to the metal species concerned. Biological mechanisms implicated in fungus survival include extracellular precipitation, complexation and crystallization, metal transformation, biosorption to cell wall, and sequestration (Fomina and Gadd, 2014). The results of Cd removal in XJ-1 under cultivation conditions with various Cd levels (1–10 mM) clearly showed that Cd principally intercepted in the tightly cell-wall bound fraction accounted for at least 80% of the metal immobilization by mycelia (**Table 1**). This fact probably indicated that retention in this compartment decreased the intracellular Cd level and acted as a vital avoidance mechanism in a primary defense system (Gadd, 1993).

**Table 1:** Cadmium (Cd) distribution in different cellular compartments of the fungus XJ-1.

Strain	Cd treated (mM)	Cd level (mg g^-1^ dry weight)		
		Tightly bound	Intracellular	Loosely bound
XJ-1	1	11.36 ± 0.11c (80.77%)	1.74 ± 0.04b (12.35%)	0.97 ± 0.05c (6.88%)
	5	54.33 ± 1.37a (86.21%)	5.33 ± 0.21a (8.46%)	4.00 ± 0.41a (5.34%)
	10	50.44 ± 0.74b (85.24%)	5.18 ± 0.55a (8.75%)	3.56 ± 0.21b (6.02%)

*Loosely bond Cd^2+^ was removed by gentle washing and strongly bound Cd^2+^ was removed by acid washing. Intracellular Cd^2+^ was not removed by washing. Data are expressed as mean ±*SD* of three independent experiments. Values within a row followed by the same letters are not significantly different (*p* > 0.05).*

**FIGURE 1:**
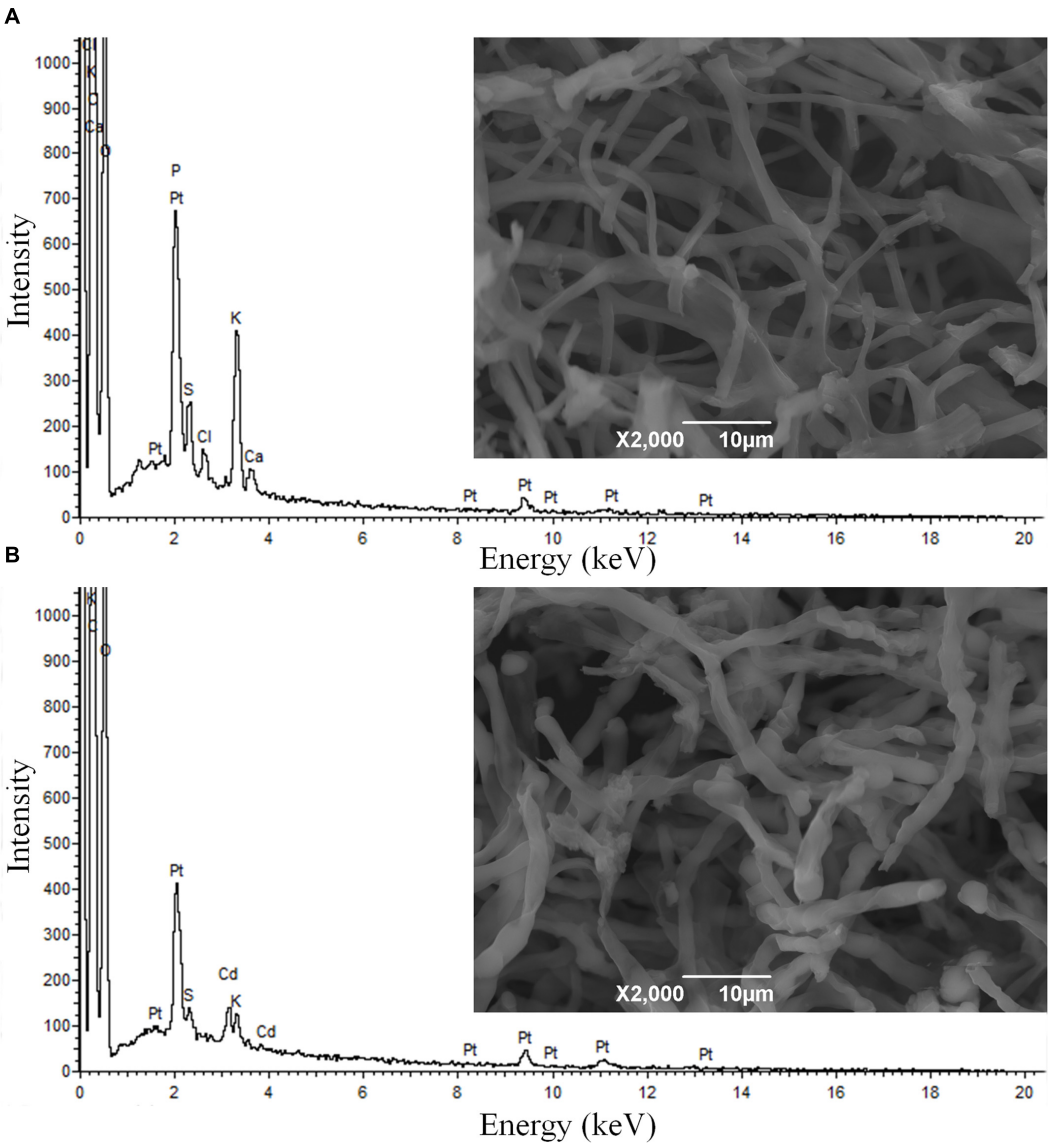
**Scanning electron microscopy (SEM) micrographs and energy dispersive X-ray (EDX) spectra of *Penicillium chrysogenum* XJ-1 cultivated **(A)** in the absence of cadmium (Cd) and **(B)** in the presence of 1 mM Cd**.

SEM micrographs show no considerable alteration in the morphology of XJ-1 hypha (**Figure 1**). SEM–EDX analysis confirmed that a large amount of Cd was bound to the cell surface of XJ-1 (Supplementary Figure S2). The fungal cell wall, as a natural barrier, is the first site of direct interaction between fungi and heavy metals (Leitão, 2009). The mechanism for the adsorption of metal ions by the cell wall does not depend on the metabolic activity of fungi, whereas the mechanism for precipitation or biosorption with excreted substances relies on the activity of cells (Gadd, 1993). Similarly, Blaudez et al. (2000) demonstrated that Cd bound to the cell wall of *P. involutus* represented a substantial fraction of the metal absorbed by its mycelia and that this binding effect might be a mechanism of this fungus to tolerate high Cd levels.

**FIGURE 2:**
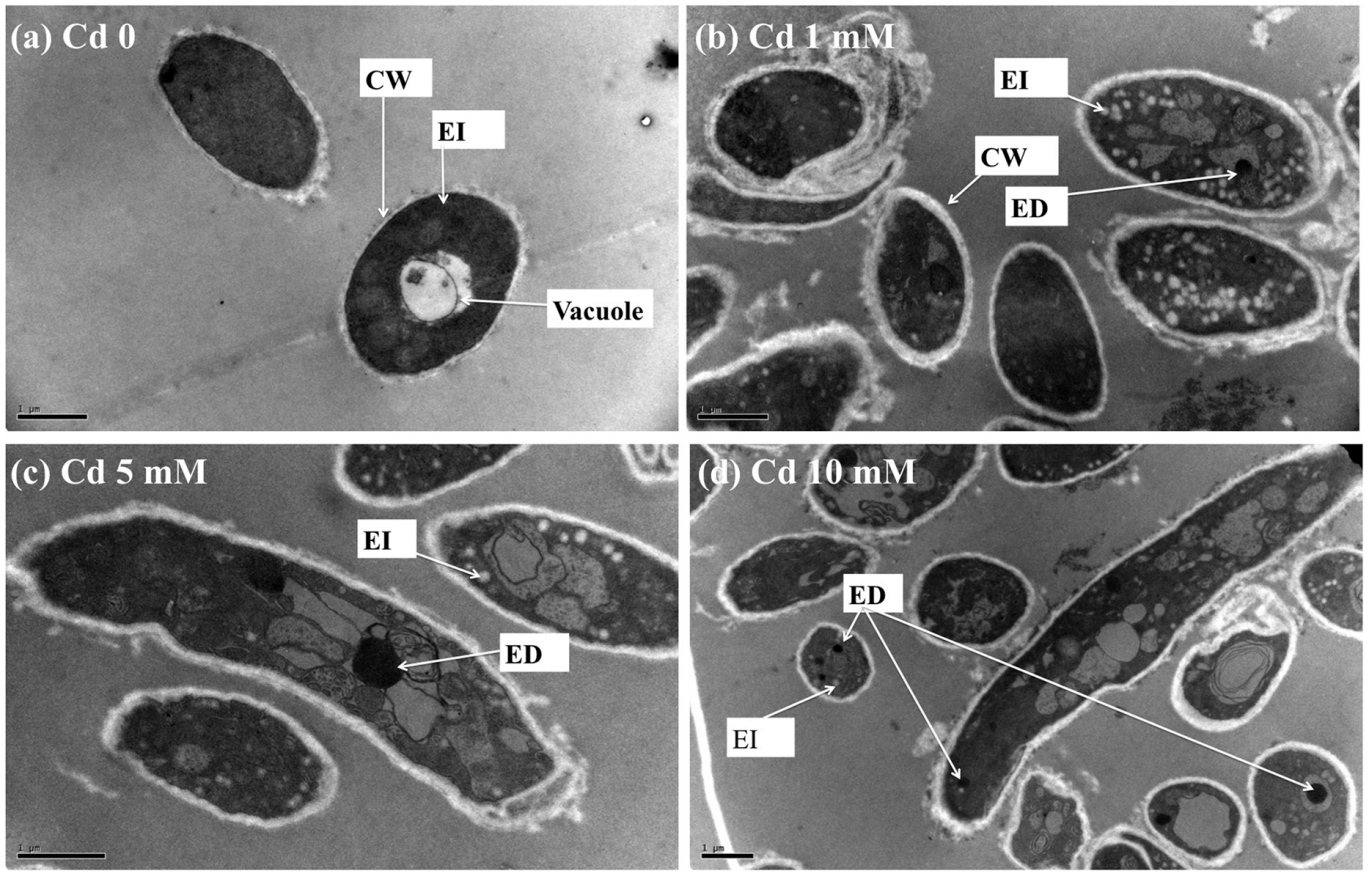
**TEM micrographs of *P. chrysogenum* XJ-1 exposed to various Cd concentrations at **(a)** 0, **(b)** 1 mM, **(c)** 5 mM, and **(d)** 10 mM for 120 h.** (CW, cell wall; EI, electron-transparent bodies; ED, electron-dense bodies; Bars represent 1 μm).

TEM images of XJ-1 grown in the presence of various Cd levels are shown in **Figure 2**. In the absence of Cd, XJ-1 showed a well-defined cellular structure and a slightly compacted cell wall (**Figure 2a**). No obvious change in morphological features of fungus XJ-1 was observed after Cd treatment (**Figures 2b–2d**). However, large amounts of electron-transparent bodies and electron-dense bodies were accumulated in the cells of XJ-1 after exposure to various Cd levels. These changes might be part of the adaptation mechanism of XJ-1 to metal toxicity according to previous results (Yang and Lin, 1998; Bianucci et al., 2011).

Intracellular Cd in fungi could be detoxified through precipitation in electron-dense bodies, such as polyphosphate granules, or through conjugation with GSH or thiol-containing compounds, such as metallothioneins (Turnau et al., 1994). In the study, GSH level was obviously increased under Cd exposure, indicating the important role of this thiol in Cd tolerance in XJ-1 (**Table 2**). Li et al. (1997) revealed that a GSH-S conjugate transporter in *Saccharomyces cerevisiae* was involved in a strictly GSH-dependent transport of Cd into the vesicles. Regardless of the mechanism of tonoplast Cd transport, the vacuolar compartmentation of Cd would limit the symplastic movement of the metal (Blaudez et al., 2000). XJ-1 may also have the above mechanism, as observed in Cd-treated fungal cells by TEM (**Figure 2**).

**Table 2:** Effects of different concentrations of Cd on the concentrations of glutathione (GSH) and oxidized glutathione (GSSG) as well as the ratio of GSH/GSSG in XJ-1.

Strain	Cd treated (mM)	GSH (μmol mg^-1^ prot)	GSSG (μmol mg^-1^ prot)	GSH/GSSG
XJ-1	0	0.99 ± 0.00d	0.25 ± 0.03a	0.40 ± 0.04c
	1	2.68 ± 0.04a	0.29 ± 0.02a	0.93 ± 0.06a
	5	2.49 ± 0.01b	0.28 ± 0.02a	0.88 ± 0.06a
	10	2.07 ± 0.09c	0.28 ± 0.02a	0.75 ± 0.04b

*Data are expressed as mean ±*SD* of three independent experiments. Values within a row followed by the same letters are not significantly different (*p* > 0.05). prot is indicative of protein.*

### Antioxidant Enzymatic Systems of XJ-1 in Response to Cd

Monitoring results of MDA concentration revealed that Cd could induce oxidative stress and damage to the membrane (Muthukumar and Nachiappan, 2010). The MDA concentration in XJ-1 cells sharply increased from 0.15 to 14.2 nmol mg^-1^ prot with the increase in Cd concentration (0, 1, 5, and 10 mM; Supplementary Figure S1). The increase in MDA concentration indicated that Cd induced lipid peroxidation and ROS generation inside the mycelia of XJ-1. Modulation of the antioxidant status is an important adaptive response to heavy metals. However, the roles of enzymatic and non-enzymatic antioxidant systems in response to heavy metals, particularly Cd, remain unclear.

**FIGURE 3:**
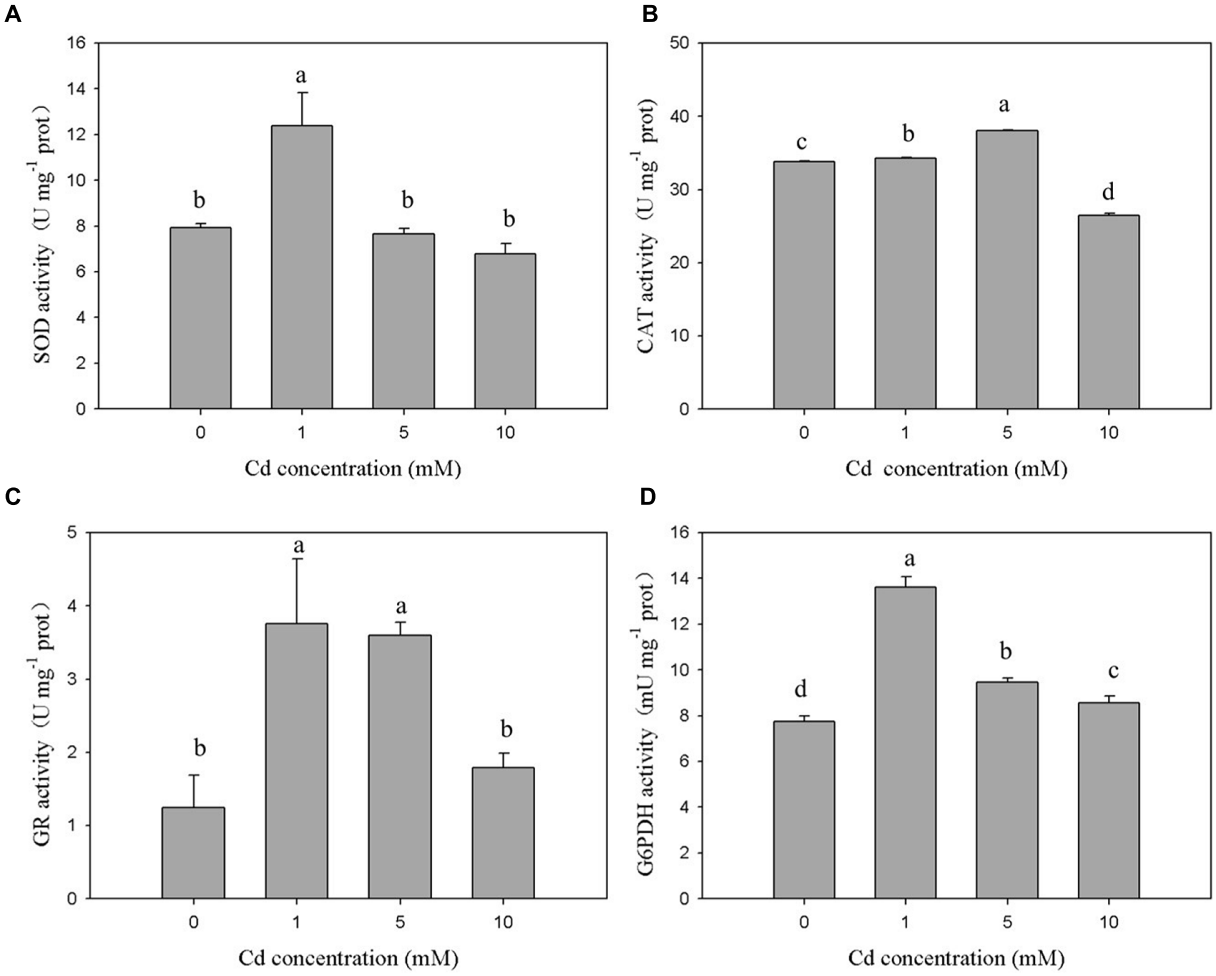
**Effects of various Cd concentrations on the activities of antioxidant enzymes in *P. chrysogenum* XJ-1 exposed to Cd for 120 h. (A)** SOD, **(B)** CAT, **(C)** GR, and **(D)** G6PDH. Bars with the same letter(s) are not significantly different at *p* > 0.05. (SOD, superoxide dismutase; CAT, catalase; GR, glutathione reductase; G6PDH, glucose-6-phosphate dehydrogenase; prot is indicative of protein).

The antioxidant enzymes of XJ-1 presented different responses to the Cd stress. As shown in **Figure 3A**, the 120-h exposure to 1 mM Cd sustainably increased SOD activity from 7.93 to 12.39 U mg^-1^. By contrast, the treatment with 5 and 10 mM Cd slightly decreased SOD activities to 7.66 and 6.78 U mg^-1^ prot, respectively. However, these changes were not significant (*p* > 0.05) when compared with the changes in the control. The exposure to 1 and 5 mM Cd increased CAT activity by 1.01 times and 1.12 times, respectively (**Figure 3B**). However, the treatment with 10 mM Cd obviously decreased (*p* < 0.05) CAT activity by 21.85% compared with the control treatment. **Figure 3C** shows that Cd markedly (*p* < 0.05) enhanced GR activity under relatively low levels of Cd (1 and 5 mM). In particular, GR activities were increased from 1.25 to 3.01 U mg^-1^ prot, 2.88, and 1.79 U mg^-1^ prot when the Cd level was increased from 0 to 1, 5, and 10 mM, respectively. Meanwhile, the activity of G6PDH (**Figure 3D**) peaked at 1 mM Cd and was significantly (*p* < 0.05) activated by Cd. G6PDH activities were 7.73, 13.62, 9.48, and 8.56 mU m^-1^ prot at 0, 1, 5, and 10 mM, respectively. These results indicated that these detected antioxidant enzymes were involved in avoiding Cd-induced oxidant stress under certain Cd levels.

In the study, Cd exerted different effects on the activities of SOD, CAT, and GSH-related enzymes in XJ-1. This finding suggested that the antioxidant system could scavenge ROS species generated by Cd at a certain range of Cd concentration and ultimately reduce the oxidative stress. The exposure to 1 mM Cd immediately increased the activities of SOD, CAT, GR, and G6PDH by 1.56, 1.01, 2.48, and 1.76 times as compared with the control treatment. Different from the activities of SOD, GR, and G6PDH, the activity of CAT peaked at 5 mM Cd. The activities of all antioxidant enzymes decreased after reaching their peaks with the increase in Cd concentrations. G6PDH activity decreased under elevated Cd levels, but its activity remained markedly higher than that in the control. The exposure to 10 mM Cd increased GR activity by 43.2%, but this increase was not significant (*p* > 0.05) when compared with the control. The different performances of the antioxidant enzymes in response to various Cd levels could be attributed to their different roles during the oxidative stress.

In aerobic organisms, SOD and CAT are crucial for cellular detoxification and controlling $O_{2}^{–}$ and H_2_O_2_ levels (Ribeiro et al., 2015). The activities of these two ROS-eliminating enzymes increased or decreased depending on metal type, metal concentration, and the tested species (Cyrne et al., 2003). In the study, the activities of both SOD and CAT initially increased and then decreased. The change trend might be caused by the co-regulation effect. Under low Cd levels, the combination of SOD and CAT efficiently blocked ROS-driven cell damage by converting them into water and molecular oxygen. The decreased activities of SOD and CAT in XJ-1 exposed to elevated Cd levels could be attributed to the inhibitory effect of the metal on enzymatic activity. GR and G6PDH, which were essential to replenish the NADPH intracellular pool, maintain cellular GSH concentration (Singh et al., 2012). Similar to the study, previous studies reported that GR and G6PDH activities increased in *Oreochromis niloticus* cells exposed to Cd (Fırat and Kargın, 2010) but decreased in *Heliscus lugdunensis* H8-2-1 and H4-2-4 exposed to 50 μM Cd (Braha et al., 2007). GR and G6PDH were involved in recycling GSSG back to GSH and contributed to the elimination of ROS and the formation of Cd–GSH complexes.

### Non-enzymatic Antioxidant Systems in XJ-1

The GSH pool is essential in cellular redox signaling and control in living organisms, including microbes (Jaeckel et al., 2005). Hence, we evaluated the GSH concentration under Cd stress in XJ-1. As listed in **Table 2**, the treatments of Cd obviously (*p* < 0.05) enhanced the GSH concentration in XJ-1 but exerted no obvious effect (*p* > 0.05) on GSSG concentration compared with the control. The GSH concentration in XJ-1 increased by 2.71 times, peaked at 1 mM Cd and then drastically decreased at the higher Cd concentrations. The GSH pool apparently decreased under the exposure to elevated external Cd concentrations but remained comparable with that in the control. Meanwhile, Cd also markedly increased the GSH/GSSG ratio. The increase of GSH/GSSG ratio represents an increased antioxidant capacity as more GSH is available.

We hypothesize that a substantial amount of GSH is removed from the GSH pool to detoxify the metal via intracellular chelation and eliminate the excessive ROS induced by Cd exposure. That is to say, GSH and its related enzymes are mainly involved in XJ-1 tolerance to Cd by maintaining cellular redox balance. Jaeckel et al. (2005) and Braha et al. (2007) reported that Cd treatment increased the GSH level in different *Heliscus lugdunensis* species. The tolerance of different fungi to heavy metals apparently involves several intracellular Cd complexes containing GSH. In *Candida glabrat*a, GSH and phytochelatin-capped CdS crystallites were found (Dameron et al., 1989). In *S. cerevisiae*, bis(glutathionato)cadmium is transported from the cytosol to the vacuole by specific proteins, such as the ATP-binding cassette (ABC) transporter YCF1, thus eventually reducing the toxicity of Cd (Li et al., 1997). In the current study, TEM microphotographs (**Figure 2**) revealed that metal sequestration might be an intracellular Cd detoxification mechanism. However, it is necessary to further investigate whether or not the increase in GSH in XJ-1 is involved in Cd chelation.

### Bioremediation of Cd-Contaminated Soil

**FIGURE 4:**
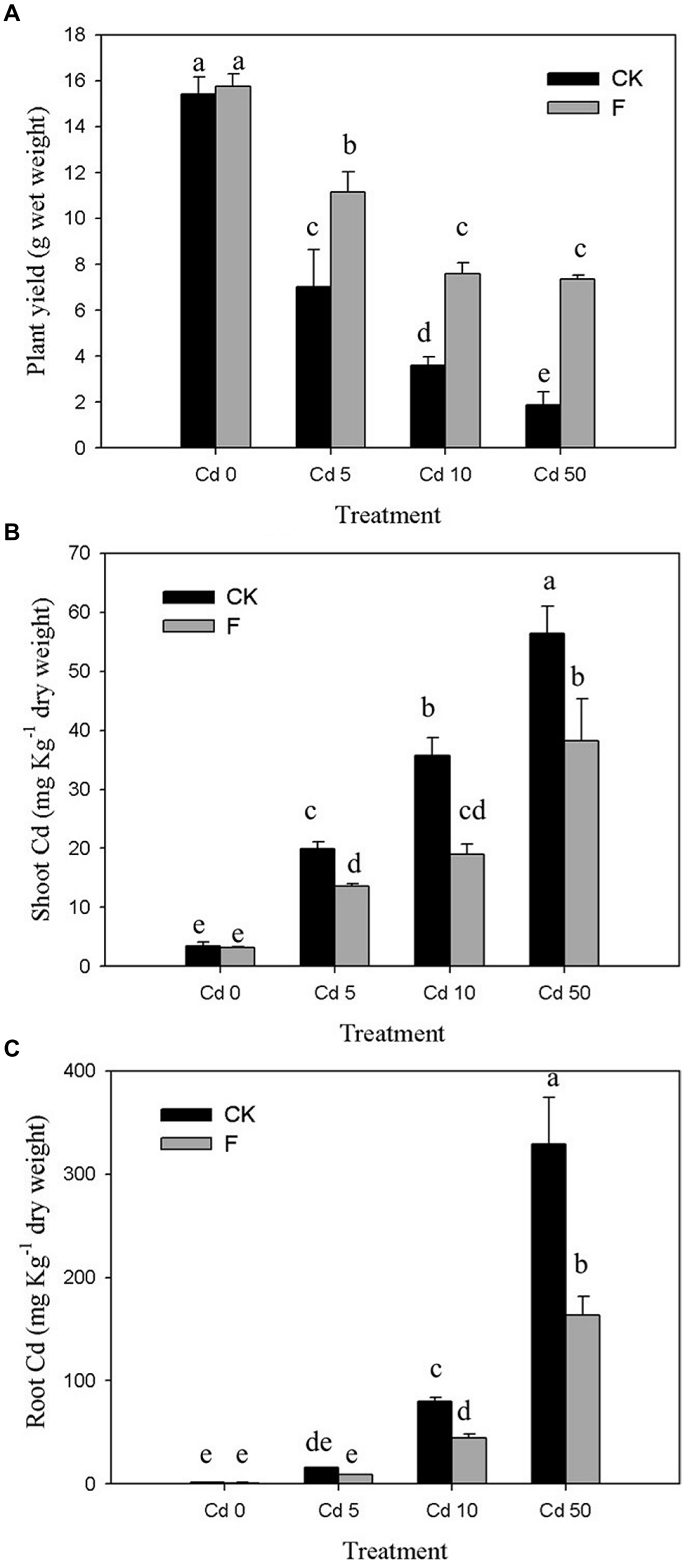
**Effects of *P. chrysogenum* XJ-1 on the plant properties of pakchoi at various Cd levels. (A)** Plant yield, **(B)** Cd uptake by plant shoots, and **(C)** Cd uptake by plant root. Bars with the same letter(s) are not significantly different at *p* > 0.05.

The results of pot experiment implied that the plant yield of pakchoi was obviously (*p* < 0.05) inhibited by Cd (**Figure 4A**). In the control group without inoculation of strain XJ-1, plant biomass was decreased by 54.42, 76.70, and 87.87% at Cd concentrations of 5, 10, and 50 mg kg^-1^ soil, respectively. The pakchoi production in the treatment group with the application of strain XJ-1 was significantly higher than that in the control group (*p* < 0.05). The shoot biomass of pakchoi in the fungal-remedied soil was 1.6–3.9 times higher than that in non-remedied soil. These results suggested that fungal application significantly promoted pakchoi growth and that this effect was pronounced at high Cd levels.

As shown in **Figure 4B**, drastic increases in Cd concentration in pakchoi shoots were determined by adding Cd. In Cd 0 soil, no obvious change was observed after inoculation of the tested fungus. For the soils contaminated with various Cd levels (5, 10, or 50 mg kg^-1^ soil), the Cd concentrations in pakchoi shoots under fungal treatment were decreased by 31.49, 47.76, and 32.24%, respectively. Similar to pakchoi shoots, pakchoi roots exhibited the enhanced Cd uptake upon Cd treatment, particularly at high Cd levels. The inoculation of the tested fungus effectively depressed Cd accumulation in plant roots by 44.21–50.26% compared with the CK groups (**Figure 4C**). The remediation effect of this microbial reagent on Cd uptake by plant shoots was remarkable in Cd 10 soils but decreased when the Cd level exceeded 10 mg kg^-1^. This phenomenon may be interpreted as follows. Since pakchoi plant is liable to Cd accumulation, the tested fungus is effective only at a relatively high degree of Cd contamination. Furthermore, this phenomenon indicates that the ability of XJ-1 to restore Cd is limited at a certain level of soil Cd because excessive Cd inhibits XJ-1 growth.

**FIGURE 5:**
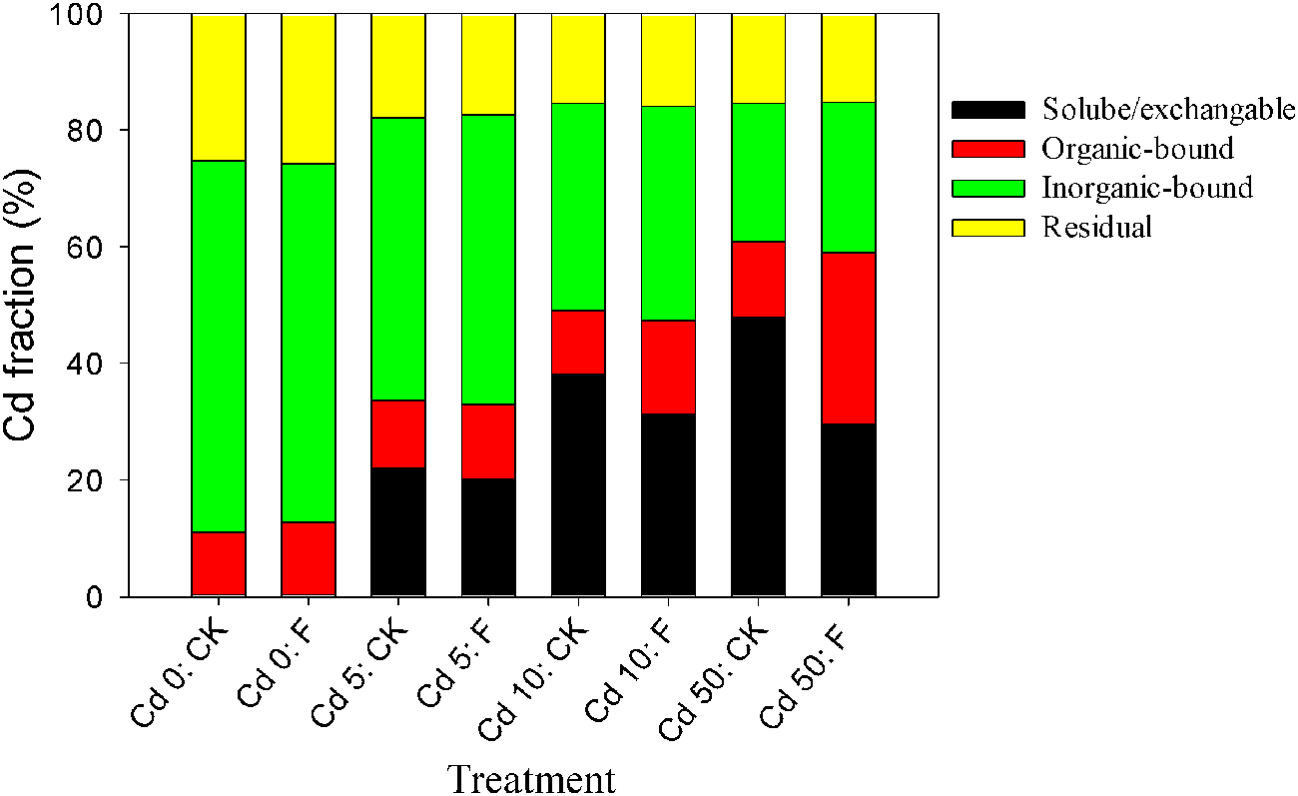
**Effects of *P. chrysogenum* XJ-1 on the distribution of Cd fractions in the soils with various Cd levels**.

The alleviation effect of the tested fungus on the toxicity of Cd to Pakchoi could be principally attributed to Cd redistribution among soil fractions after soil microbial remediation. The percentages of various Cd fractions in the soils are presented in **Figure 5**. In the soil without fungal inoculation, the soluble/exchangeable Cd increased from 0.28 to 47.98% with the increase in the Cd level. Fungal reagent application redistributed Cd among fractions at different Cd levels. In all Cd treatments (Cd 0, Cd 1, Cd 5, and Cd 10), fungal inoculation, respectively, reduced soluble/exchangeable Cd by 11.70, 8.98, 18.16, and 38.18%, and promoted organic-bound Cd by 1.15, 1.11, 1.47, and 2.28 times compared with the control, respectively. Inorganic-bound and residual fractions with fungal amendments showed no significant changes (*p* > 0.05). These results demonstrated that the inoculation of XJ-1 effectively converted Cd forms in these artificial Cd-contaminated soils and that the bioavailable Cd was mainly transferred to the organic-bound fraction. The possible mechanism is that the mobile Cd is tightly absorbed on the cell walls and accumulated in the mycelia of XJ-1.

The conversion of Cd from bioavailable fractions into inert species is important in governing the uptake of Cd by plants. The ability of microorganisms to immobilize heavy metals introduced into soils had been proposed by Xu et al. (2012a) and Wang et al. (2012). Microbes affect metal immobilization in three aspects: biosorption of metals on the cell walls via ion exchange, chelation, adsorption, and extracellular precipitation, intracellular uptake of metals, and transformation of metal species (Gadd, 1993; Sing, 2006; Xu et al., 2012b). In this study, the significant role of XJ-1 in reducing bioavailable Cd in soils can be primarily ascribed to its capacities of extracellular biosorption and intracellular sequestration. Our previous studies demonstrated that the cell wall of XJ-1 greatly contributed to the adsorption of Cd ions and that the –OH and –C=O groups on the cell surface were responsible for Cd binding on fungal cells (Xu et al., 2012b). Moreover, the results in the current study (**Table 1** and **Figure 2**) revealed that mobile Cd was partly accumulated and sequestered in XJ-1 cells.

## Conclusion

*Penicillium chrysogenum* XJ-1 can endure high levels of Cd contamination via Cd detoxification mechanisms, such as biosorption, metal sequestration, and antioxidant defense systems. XJ-1 can colonize Cd-polluted soils, promote pakchoi production, and reduce bioavailable soil-Cd fractions via high-affinity biosorption on fungal biomass. However, this fungus cannot directly enhance plant growth. Hence, XJ-1 as a metal immobilizing agent may be combined with other soil amendments (compost and biochar), which might generate an econiche to strengthen the growth of XJ-1. The combined effect still requires further investigation.

## Author contributions

QH and WC designed the research, analyzed data, critically reviewed the manuscript, and contributed reagents/materials/analysis tools. XX performed research, analyzed data, and wrote the paper. LX, WZ, and ZZ performed research and analyzed data.

## Conflict of Interest Statement

The authors declare that the research was conducted in the absence of any commercial or financial relationships that could be construed as a potential conflict of interest.

## References

[B1] Bai Z. Harvey L. M. McNeil B. (2003). Oxidative stress in submerged cultures of fungi. *Crit. Rev. Biotechnol.* 23 267–302. 10.1080/07388550390449294 15224892

[B2] Bianucci E. Fabra A. Castro S. (2011). Involvement of glutathione and enzymatic defense system against cadmium toxicity in *Bradyrhizobium* sp. Strains (peanut symbionts). *Biometals* 25 23–32. 10.1007/s10534-011-9480-z 21766174

[B3] Blaudez D. Botton B. Chalot M. (2000). Cadmium uptake and subcellular compartmentation in the ectomycorrhizal fungus *Paxillus involutus*. *Microbiology* 146 1109–1117. 10.1099/00221287-146-5-1109 10832638

[B4] Braha B. Tintemann H. Krauss G. Ehrman J. Bärlocher F. Krauss G. J. (2007). Stress response in two strains of the aquatic hyphomycete *Heliscus lugdunensis* after exposure to cadmium and copper ions. *Biomteals* 20 93–105. 10.1007/s10534-006-9018-y 16900400

[B5] Chen H. S. Huang Q. Y. Liu L. N. Cai P. Liang W. Li M. (2010). Poultry manure compost alleviates the phytotoxicity of soil cadmium: influence on growth of pakchoi (*Brassica chinensis* L.). *Pedosphere* 20 63–70. 10.1016/S1002-0160(09)60283-6

[B6] Cyrne L. Martins L. Fernandes L. Marinho H. S. (2003). Regulation of antioxidant enzymes gene expression in the yeast *Saccharomyces cerevisiae* during stationary phase. *Free Radic. Biol. Med.* 34 385–393. 10.1016/S0891-5849(02)01300-X 12543254

[B7] Dameron C. T. Reese R. N. Mehra R. K. Kortan A. R. Carroll P. J. Steigerwald M. L. (1989). Biosynthesis of cadmium sulphide quantum semiconductor crystallites. *Nature* 338 596–597. 10.1038/338596a0

[B8] Deng Z. Zhang R. Yang S. Hu L. Tan H. Cao L. (2014). Characterization of cd-, pb-, zn-resistant endophytic *Lasiodiplodia* sp. mxsf31 from metal accumulating portulaca oleracea and its potential in promoting the growth of rape in metal-contaminated soils. *Environ. Sci. Pollut. Res.* 21 2346–2357. 10.1007/s11356-013-2163-2 24062066

[B9] Fırat Ö Kargın F. (2010). Effects of Zinc and cadmium on erythrocyte antioxidant systems of a freshwater fish *Oreochromis niloticus*. *J. Biochem. Mol. Toxicol.* 24 223–229. 10.1002/jbt.20327 20143450

[B10] Fomina M. Gadd G. M. (2014). Biosorption: current perspectives on concept, definition and application. *Bioresour. Technol.* 160 3–14. 10.1016/j.biortech.2013.12.102 24468322

[B11] Gadd G. M. (1993). Interaction of fungi with toxic metals. *New Phytol.* 124 25–60. 10.1111/j.1469-8137.1993.tb03796.x

[B12] Gadd G. M. (2001). “Metal transformations,” in *Fungi in Bioremediation* ed. Gadd G. M. (Cambridge: Cambridge University Press) 359–382.

[B13] Hartley J. Cairney J. W. G. Meharg A. A. (1997). Do ectomycorrhizal fungi exhibit adaptive tolerace to potentially toxic metals in the environment? *Plant Soil* 189 303–319. 10.1023/A:1004255006170

[B14] Herrero E. Ros J. Bellí G. Cabiscol E. (2008). Redox control and oxidative stress in yeast cells. *Biochim. Biophys. Acta* 1780 1217–1235. 10.1016/j.bbagen.2007.12.004 18178164

[B15] Jaeckel P. Krauss G. Menge S. Schierhorn A. Rücknagel P. Krauss G. J. (2005). Cadmium induces a novel metallothionein and phytochelatin 2 in an aquatic fungus. *Biochem. Biophys. Res. Commun.* 333 150–155. 10.1016/j.bbrc.2005.05.083 15939401

[B16] Janoušková M. Pavlíková D. (2010). Cadmium immobilization in the rhizosphere of arbuscular mycorrhizal plants by the fungal extraradical mycelium. *Plant Soil* 332 511–520. 10.1007/s11104-010-0317-2

[B17] Lanfranco L. (2007). The fine-tuning of heavy metals in mycorrhizal fungi. *New Phytol.* 174 3–6. 10.1111/j.1469-8137.2007.02029.x 17335491

[B18] Leitão A. L. (2009). Potential of *Penicillium* species in the bioremediation field. *Int. J. Environ. Res. Public Health* 6 1393–1417. 10.3390/ijerph6041393 19440525 PMC2681198

[B19] Li Z. S. Lu Y. P. Zhen R. G. Szczypka M. Thiele D. J. Rea P. A. (1997). A new pathway for vacuolar cadmium sequestration in *Saccharomyces cerevisiae*: YCF1-catalyzed transport of bis(glutathionato)cadmium. *Proc. Natl. Acad. Sci. U.S.A.* 94 42–47. 10.1073/pnas.94.1.42 8990158 PMC19233

[B20] Muthukumar K. Nachiappan V. (2010). Cadmium-induced oxidative stress in *Saccharomyces cerevisiae*. *Indian J. Biochem. Biophys.* 47 383–387. 21355423

[B21] Ott T. Fritz E. Polle A. Schützendübel A. (2002). Characterisation of antoxidative systems in the ecomycorrhiza-building basidiomycete *Paxillus involutus* (Bartsch) Fr. and its reaction to cadmium. *FEMS Microbiol. Ecol.* 42 359–366. 10.1111/j.1574-6941.2002.tb01025.x 19709295

[B22] Pócsi I. Prade R. A. Penninckx J. (2004). Glutathione, altruistic metabolite in fungi. *Adv. Microb. Physiol.* 49 1–76. 10.1016/S0065-2911(04)49001-8 15518828

[B23] Postma E. Verduyn C. Scheffers W. A. Van Dijken J. P. (1989). Enzymic analysis of the Crabtree effect in glucose-limited chemostat cultures of *Saccharomyces cerevisiae*. *Appl. Environ. Microbiol.* 55 468–477. 2566299 10.1128/aem.55.2.468-477.1989 PMC184133

[B24] Purchase D. Miles R. J. Young T. W. K. (1997). Cadmium uptake and nitrogen fixing ability in heavy metal resistant laboratory and field strains of *Rhizobium leguminosarum* biovar trifolii. *FEMS Microbiol. Ecol.* 22 85–93. 10.1111/j.1574-6941.1997.tb00359.x

[B25] Ribeiro T. P. Fernandes C. Melo K. V. Ferreira S. S. Lessa J. A. Franco R. W. A. (2015). Iron, copper, and manganese complexes with in vitro superoxide dismutase and/or catalase activities that keep *Saccharomyces cerevisiae* cells alive under severe oxidative stress. *Free Radic. Biol. Med.* 80 67–76. 10.1016/j.freeradbiomed.2014.12.005 25511255

[B26] Ruiz J. M. Blumwald E. (2002). Salinity-induced glutathione synthesis in *Brassica napus*. *Planta* 214 965–969. 10.1007/s00425-002-0748-y 11941474

[B27] Sing H. (2006). *Mycoremediation.* Hoboken, NJ: John Wiley & Sons, Inc.

[B28] Singh S. Anand A. Srivastava P. K. (2012). Regulation and properties of glucose-6-phosphate dehydrogenase: a review. *Int. J. Plant Physiol. Biochem.* 4 1–19.

[B29] Siripornadulsil S. Siripornadulsil W. (2013). Cadmium-tolerant bacteria reduce the uptake of cadmium in rice: potential for microbial bioremediation. *Ecotoxicol. Environ. Saf.* 94 94–103. 10.1016/j.ecoenv.2013.05.002 23731867

[B30] Sposito G. Lund J. L. Chang A. C. (1982). Trace metal chemistry in arid-zone field soils amended with sewage sludge: I. Fractionation of Ni, Cu, Zn, Cd, and Pb in solid phases. *Soil Sci. Soc. Am. J.* 46 260–264. 10.2136/sssaj1982.03615995004600020010x

[B31] Turnau K. Kottke I. Dexheimer J. Botton B. (1994). Element distribution in mycelium of *Pisolithus arrhizus* treated with cadmium dust. *Ann. Bot.* 74 137–142. 10.1006/anbo.1994.1103

[B32] Wang F. Y. Wang L. Shi Z. Y. Li Y. J. Song Z. M. (2012). Effects of AM inoculation and organic amendment, alone or in combination, on growth, P nutrition, and heavy-metal uptake of tobacco in Pb-Cd-contaminated soil. *J. Plant Growth Regul.* 31 549–559. 10.1007/s00344-012-9265-9

[B33] Xu P. Liu L. Zeng G. Huang D. Lai C. Zhao M. (2014). Heavy metal-induced glutathione accumulation and its role in heavy metal detoxification in phanerochaete chrysosporium. *Appl. Microbiol. Biotechnol.* 98 6409–6418. 10.1007/s00253-014-5667-x 24723291

[B34] Xu X. Huang Q. Huang Q. Chen W. (2012a). Soil microbial augmentation by an EGFP-tagged *Pseudomonas putida* X4 to reduce phytoavailable cadmium. *Int. Biodeterior. Biodegradation* 71 55–60. 10.1016/j.ibiod.2012.03.006

[B35] Xu X. Xia L. Huang Q. Gu J. D. Chen W. (2012b). Biosorption of cadmium by a metal-resistant filamentous fungus isolated from chicken manure compost. *Environ. Technol.* 33 1661–1670. 10.1080/09593330.2011.641591 22988626

[B36] Yang F. Lin L. (1998). Cytostructure, lipopolysaccharide, and cell proteins analysis from *Rhizobium fredii*. *Bot. Bull. Acad. Sin.* 39 261–267.

[B37] Zafar M. N. Nadeem R. Hanif M. A. (2007). Biosorption of nickel from protonated rice bran. *J. Hazard. Mater.* 143 478–485. 10.1016/j.jhazmat.2006.09.055 17049420

[B38] Zhang X. H. Zhu Y. G. Chen B. D. Lin A. J. Smith S. E. Smith F. A. (2005). Arbuscular mycorrhizal fungi contribute to resistance of upland rice to combined metal contamination of soil. *J. Plant Nutr.* 28 2065–2077. 10.1080/01904160500320871

