# Supplementary material for: Role of Penicillium chrysogenum XJ-1 in the Detoxification and Bioremediation of Cadmium
Source: Front Microbiol. 2015 Dec 21;6:1422. doi: 10.3389/fmicb.2015.01422 (PMC4685053; doi:10.3389/fmicb.2015.01422)
Supplement: Supplementary file 1 [file Data_Sheet_1.DOCX]

**Role of *Penicillium chrysogenum* XJ-1 in the detoxification and bioremediation of cadmium**

Xingjian Xu^1,3^, Lu Xia^1^, Wei Zhu^1^, Zheyi Zhang^1^, Qiaoyun Huang^1,2*^ and Wenli Chen^1*^

^1^State Key Laboratory of Agricultural Microbiology, Huazhong Agricultural University, Wuhan 430070, China

^2^Key Laboratory of Arable Land Conservation (Middle and Lower Reaches of Yangtze River), Ministry of Agriculture, College of Resources and Environment, Huazhong Agricultural University, Wuhan 430070, China

^3^Northeast Institute of Geography and Agroecology, Chinese Academy of Sciences, Changchun 130102, China

*Corresponding author: Wenli Chen, Qiaoyun Huang, State Key Laboratory of Agricultural Microbiology, Huazhong Agricultural University, Wuhan 430070, China

Email: [wlchen@mail.hzaui.edu.cn](mailto:wlchen@mail.hzaui.edu.cn), [qyhuang@mail.hzau.edu.cn](mailto:qyhuang@mail.hzau.edu.cn)

Phone: +86-27-87671033

Fax: +86-27-87280670

**Supporting Information**

**Fig. S1** Effects of various concentrations of Cd on the MDA concentration in *P. chrysogenum* XJ-1. Bars with the same letter(s) are not significantly different at *p* > 0.05.

**Fig. S2** Elemental spectra of *P. chrysogenum* XJ-1 cultivated (a) in the absence of Cd and (b) in the presence of 1 mM Cd. Red color: Cd; Green color: other elements

**Fig. S1**


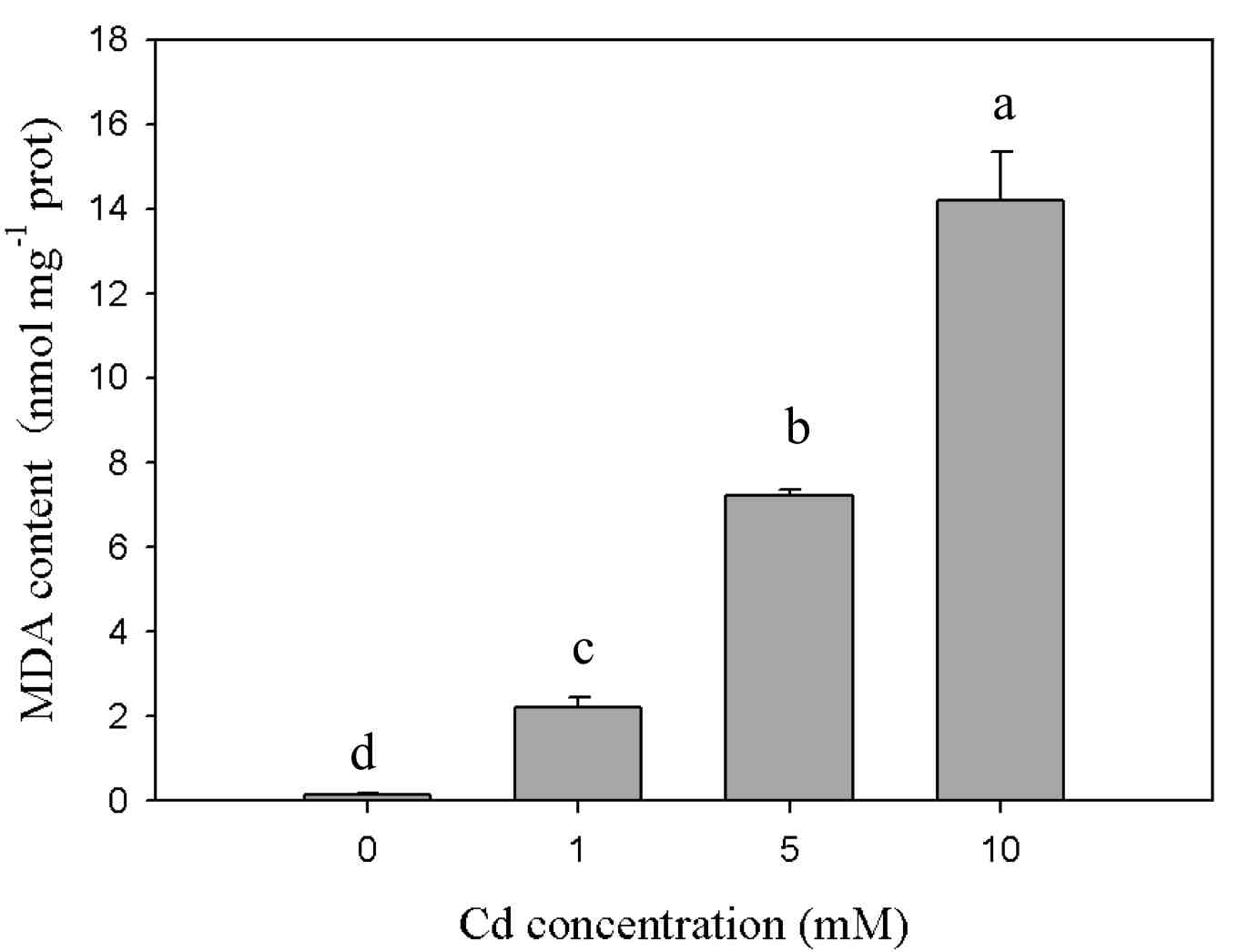


**Fig. S2**

**
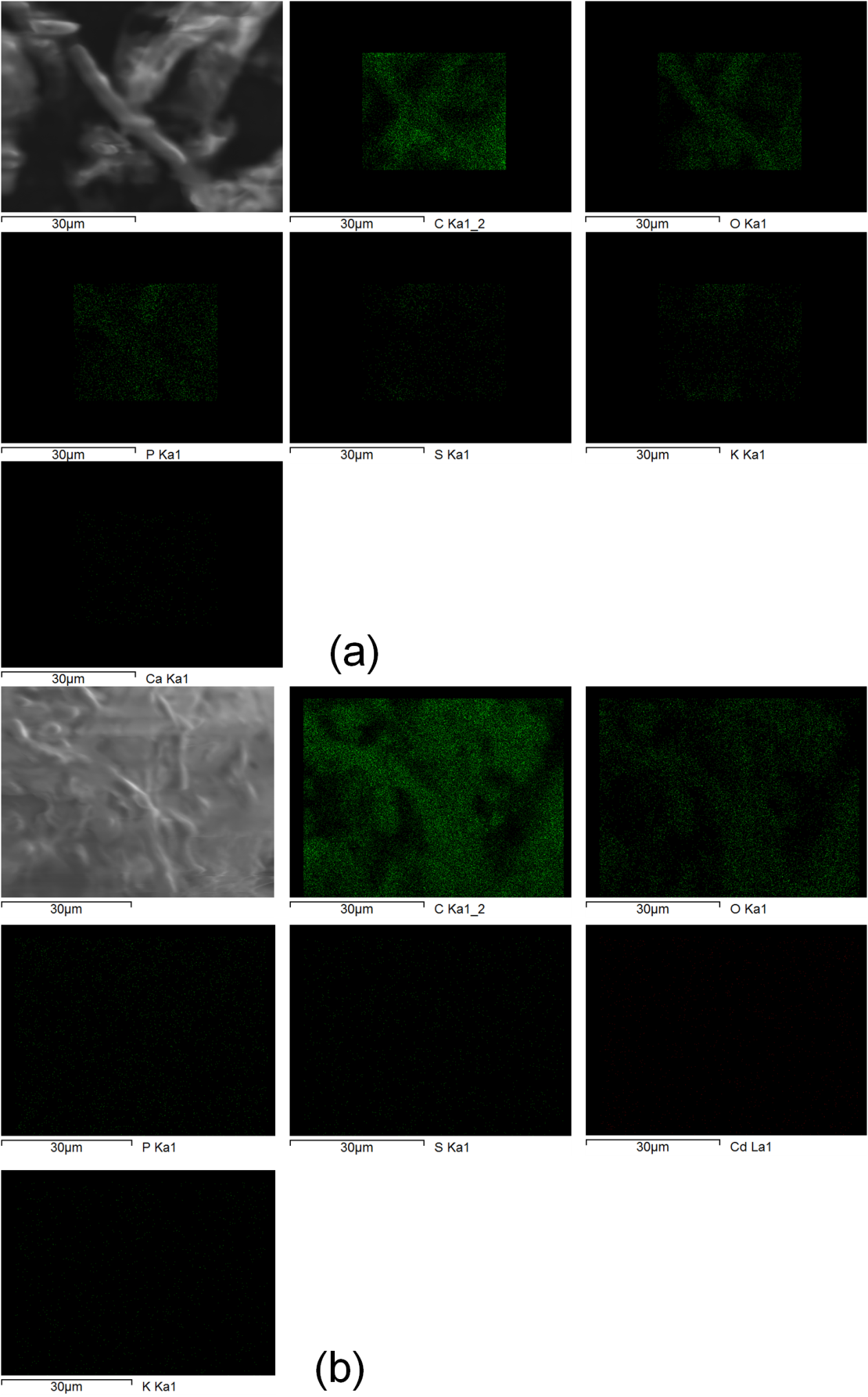
**
